# Heavy-atom tunnelling in Cu(ii)N_6_ complexes: theoretical predictions and experimental manifestation†

**DOI:** 10.1039/d0sc00160k

**Published:** 2020-02-18

**Authors:** Itzhak Sedgi, Sebastian Kozuch

**Affiliations:** Department of Chemistry, Ben-Gurion University of the Negev Beer-Sheva 841051 Israel kozuch@bgu.ac.il; Department of Analytical Chemistry, Nuclear Research Center Negev PO Box 9001 Beer-Sheva Israel

## Abstract

The degenerate rearrangement on Jahn–Teller distorted metal complexes is a promising reaction for the observation of significant heavy atom quantum mechanical tunnelling. Herein, a family of Cu(ii)–N_6_ complexes are theoretically proven to exhibit rapid dynamical Jahn–Teller tunneling even close to the absolute zero. The manifestation of our predictions apparently appeared in solid state EPR experimental measurements on [Cu(en)_3_]SO_4_ more than 40 years ago, without the authors realizing that it was a quantum outcome.

## Abstract 2

The degenerate rearrangement on Jahn–Teller distorted metal complexes is a promising reaction for the observation of significant heavy atom quantum mechanical tunnelling.

## Graphical abstract

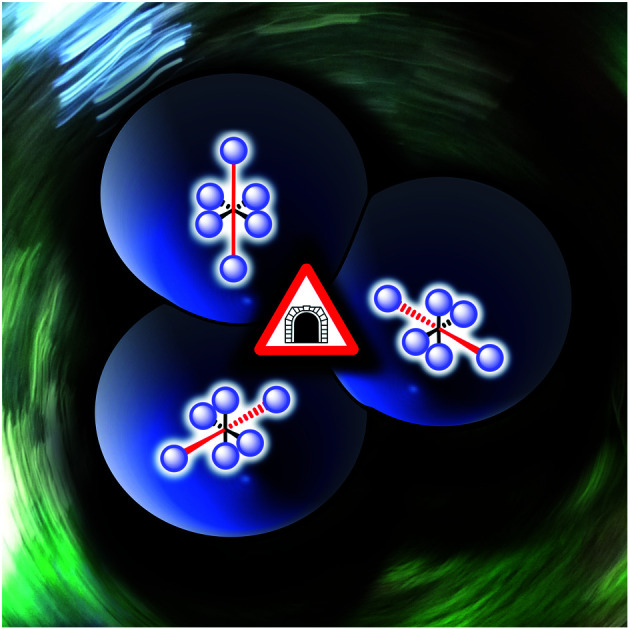

## Introduction

**Fig. 1:**
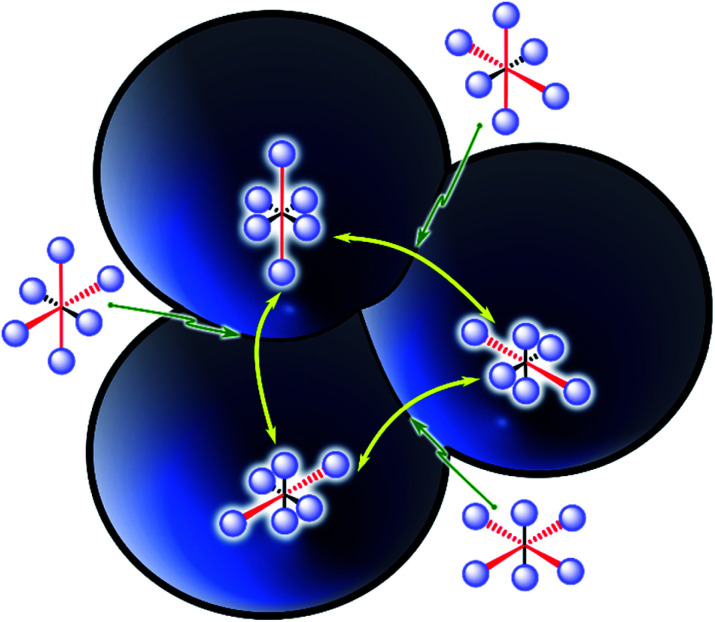
The degenerate triple-well potential energy surface of a typical d^9^, 21 e-complex, including the six tetragonal Jahn–Teller distortions from the octahedron. The elongated geometries (with antibonding 
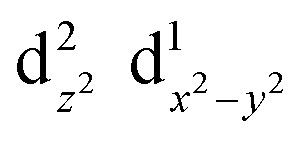
 occupation generating two long and four short bonds) form the three local minima, while the compressed geometries (
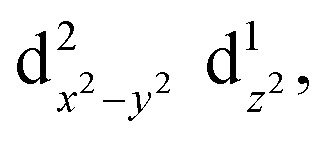
 with two short and four long bonds) act as transition states for the degenerate rearrangements between the previous states. Yellow arrows indicate the three possible rearrangements. Red and black arrows indicate the long and short coordination bonds, respectively. The fully symmetrical O_h_ geometry would be at the centre, as a third-order saddle point.

The Jahn–Teller effect^1,2^ (JTE) predicts that a non-linear system with degenerate electronic states will distort in order to lift the degeneracy and lower its energy. In many cases, the distortion leads to a set of similar isoenergetic isomers, generating a “multi-well” degenerate potential energy surface. In Cu(ii) octahedral complexes, possibly the most studied compounds of this type,^3,4^ the JTE leads to tetragonal distortions due to a breakage of the degeneracy of the antibonding e_g_ orbitals. This forms “elongated” and “compressed” geometries, generating a multi-well system known as the “warped Mexican hat” (Fig. 1).^3,5–9^ The nature of the JT distortion (elongated, with antibonding 
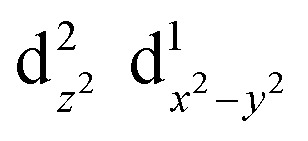
 occupation, or compressed, 
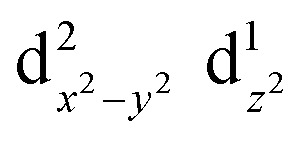
) cannot be easily predicted, and both geometries are theoretically valid for the first order.^3,5,10^ However, with six identical ligands (homoleptic complex) the elongated form will always be energetically favourable due to pseudo-JT correction^5^ (even in non-homoleptic complexes the compressed form is rarely observed).^3^

The interconversion between these isomers occurs with relative ease *via* a compressed geometry transition state (Fig. 1). Low barriers allow a rapid transition (high automerization frequency in a “dynamic” JT distortion), while high activation energy supposedly hinders the reaction (“static” JT), especially at low temperatures. However, an alternative path to the over-the barrier automerization exists even close to the absolute zero, consisting of quantum mechanical tunnelling (QMT) driven dynamic JT.

The role of heavy atom QMT^11–13^ in molecular systems (that is, any atom heavier than H or He) has been studied since the early 80's, starting with the degenerate π-bond shifting of cyclobutadiene.^14^ Since then, many other degenerate rearrangements have been seen to react by a QMT mechanism.^15–22^ As any distortion lowers the symmetry of a molecule, all of these reactions are driven by a double (or multi) well potential energy surface created due to the different flavors of the JTE.^2^ And in all of these reactions, the fast tunnelling rate is caused by the small particle mass (actually, the small reduced mass of the system in the reaction coordinate), the relatively low barrier height, and most critically, the narrow tunnelling distance.^23^ Common experimental indications of tunneling are a high kinetic isotope effect (KIE) and temperature independent rates (producing negligible Arrhenius activation energies and low pre-activation factors).^23,24^

In contrast, QMT dynamic JT in solid state systems was already proposed^25,26^ by Bersuker in 1963 and later confirmed experimentally mostly by cryogenic EPR detection of tunnelling splitting (3Γ) in solid solutions of JT active centres in insulators (such as Cu^2+^ doped MgO^2,9,27–33^). These systems have seen a revival due to their potential use in quantum computers, colossal magnetoresistance, and even in superconductivity.^32^ It is worth noting that solid and gas phase chemistry bear completely different surrounding conditions. Crystal structures are dominated by strong pressures and interactions (including counterion effects^34^) that might force the complexes to stay in a defined, static isomer. But in JT systems where the atomic displacement is short (measured here as the JT radius, see below) and with almost insignificant chemical changes, the crystal pressure actually enhances the QMT dynamics,^32^ as it constrains the atomic trajectories. This, combined with the fact that the rearrangement barriers for Cu oxides are radically low (of the order of one kJ mol^−1^), makes the tunnelling close to the absolute zero extremely probable. In fact, due to such low activation energies, the EPR study has to be carried out at extremely low temperatures (close to 1 K), to distinguish the QMT and classical dynamic JTE, and to avoid other dynamical effects.

The also known nitrogen based Cu(ii) complexes are tougher JT systems.^7,8,34,35^ Although it was speculated that QMT might play a role in the dynamic JTE of such complexes (specifically in Cu(ii)-doped hexaimidazole in a Zn(ii) matrix^36^ at 77 K), later on these observations were disproved.^37–40^ “Genuine” dynamic JT has been seen in many of these crystal cases (as seen in the temperature dependency of Cu–N bond lengths), but tunnelling from the ground state for Cu(ii)–N_6_ complexes seemed to be, apparently, impossible.

Herein we present computational evidence of the crucial role of QMT during the degenerate rearrangement of Cu(ii)–N_6_ type complexes in the gas phase under cryogenic conditions (which simulates the experimental results that can be obtained in supersonic expansion techniques,^41^ co-deposition with noble gas weakly interacting matrices,^19,42^ or even in He nanodroplets^43^). Even if not recognized at that time, this effect can actually be seen in a long-standing solid state EPR experiment.^34^

## Methods

All the automerization rate constants were computed with semi-classical canonical variational theory (CVT)^44^ adding accurate multidimensional tunnelling correction with the small curvature tunnelling (SCT) method^45,46^ (including quantized reactant state tunnelling – QRST^47^ only at low temperatures, and with a small step size of 0.001 bohr, see the ESI†). In heavy atom QMT severe corner-cutting is uncommon, and the relatively small differences between ZCT and SCT values (approximately an order of magnitude) justify the use of the latter without requiring large curvature tunnelling corrections. DFT computations were carried out with Gaussian 16,^48^ while the rate constants were computed with Polyrate 17,^49^ with Gaussrate 17B^50^ as an interface to Gaussian.

**Fig. 2:**
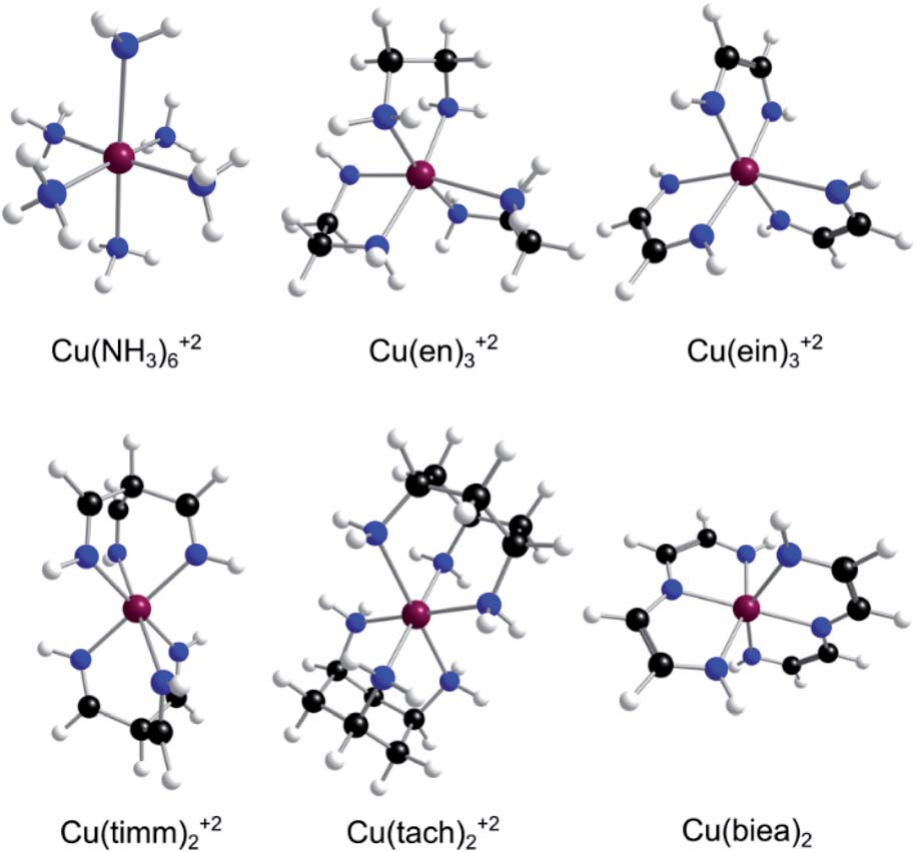
The family of Cu(ii)–N_6_ amine and imine complexes with mono-, bi-, and tri-denticity variance [en^34^ = ethanediamine, ein = ethanediimine timm = “tripodal” tris(iminomethyl)methane, tach^35^ = “tripodal” 1,3,5-triaminocyclohexane, and biea = “pincer” bis(iminoethylidyne)azanide]. Cu(biea)_2_ is formally a ML_4_X_2_ neutral complex with only two possible isomers and one transition state (in contrast to all the other ML_6_^2+^ systems containing three minima and three first-order saddle points).

Since QMT computations are highly demanding, a fast functional and basis set combination was carefully chosen after a benchmark procedure on the activation energy of Cu(ii) systems with en, ein, NH_3_, biea and timm ligands (see Fig. 2). For the reference energies we used DLPNO-CCSD(T)/aug-cc-pVQZ//MN15/Def2-TZVPD with tight PNO criteria (computed with ORCA 4.0).^51–55^ This method is not foolproof, but is orders of magnitude more reliable than any DFT scheme.^51^ No severe static correlation was found using the T1 and the % TAE(T) diagnostics,^56,57^ and negligible differences were found between the highest PNO levels (see the ESI†), justifying the selected reference method (especially considering the impossibility of using canonical CCSD(T) with complete basis set schemes on larger molecules). From all the functionals and basis sets tested, we found the PBE0/6-31+G(d) method to be the most accurate while still being relatively fast (see Tables S1–S3 in the ESI†).^58^

We acknowledge that even with the high-level SCT tunnelling method and the selection of the functional through careful benchmarking, small errors in the geometries and energies can lead to exponentially large errors in the computation of the rate constants. Therefore, the presented results are not to be taken at face value. Still, our predictions and conclusions stand as semi-quantitative values, possibly within an accuracy of one or two orders of magnitude. KIE computations are also sensitive, but they take advantage of error cancellation in the ratio of rates between isotopologues.

We must point out that even if computational results are not highly accurate, cryogenic experimental tests are also extremely sensitive to the technique (be it gas phase measurements through supersonic expansion, co-deposition with noble gases, solid-state doped complexes, a liquid state in He droplets, or any other low temperature available method). Therefore, a direct comparison between experiment and computation (or between experiment and experiment!) must be done taking all these reproducibility issues into consideration.^42,59^

## Results and discussion

Most Cu(ii) systems that experience QMT in solid matrices are oxygen based, where the metal–ligand bond strength is weak and therefore the automerization of JT structures is exceedingly easy. For this study, we sought complexes with stronger bonds, with higher rearrangement activation energies and lower probability of reaction by a classical over-the-barrier mechanism. Nitrogen-based complexes (amines and imines), being relatively strong Lewis bases, proved to be a much better choice than oxygenated systems, even if the barriers are still low (circa 6 to 9 kJ mol^−1^, see Table 1). Therefore, based on common ligands, we selected six 21 e^−^ Cu(ii) complexes that show a well-defined JTE (Fig. 2). These mono-, bi-, and tri-dentate amine and imine complexes permitted us to explore the variability caused by ligand denticity and N-hybridization.

**Table 1:** JT radius (Å, eqn (1)), activation energy (kJ mol^−1^), semi-classical (CVT) and QMT included (SCT) rate constants (s^−1^), and ^14^N/^15^N KIE (all nitrogens substituted), at 4 K, of the six complexes described in Fig. 2

Complex	*R*_JT_	Δ*E*^‡^	*k*_CVT_	*k*_SCT_	KIE
Cu(NH_3_)_6_^2+^	0.56	8.1	9 × 10^−88^	9 × 10^−2^	2.5
Cu(ein)_3_^2+^	0.45	7.3	2 × 10^−71^	8 × 10^2^	1.8
Cu(en)_3_^2+^	0.47	6.4	5 × 10^−63^	1 × 10^3^	1.5
Cu(tach)_2_^2+^	0.42	5.8	4 × 10^−50^	4 × 10^4^	2.1
Cu(timm)_2_^2+^	0.50	9.4	2 × 10^−101^	2 × 10^−1^	2.2
Cu(biea)_2_	0.38	7.8	3 × 10^−75^	2 × 10^4^	1.9

We computed the automerization rates for the Cu(ii)–N_6_ complexes from 4 to 400 K using the CVT semi-classical method, adding the SCT tunnelling correction computed at the benchmarked PBE0/6-31+G(d) level, as described in the Methods section. The distortion was gauged according to the JT radius (*R*_JT_, eqn (1)),^7^ where Δ*d*_*i*_ is the bond length difference between the average and the *i*'th M–L bonds,1
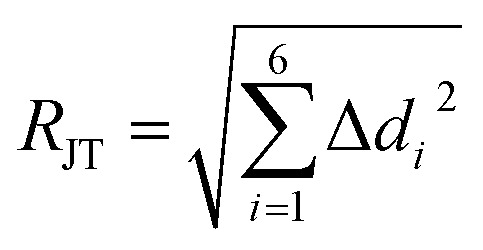


This measure is similar to the effective trajectory of a QMT process,^17,60,61^ and therefore their low values (combined with the low activation energies, see Table 1), suggest a high QMT probability. Solid-state experimental *R*_JT_ seems to be shorter than our gas phase values (for example, 0.33 *vs.* 0.42 Å for Cu(tach)_2_^2+^) due to the crystal pressure (see above),^35^ which points to a significantly faster QMT rate for the former. Still, as can be seen in Table 1, even if the results with nitrogen-based ligands in the gas phase are much slower than copper oxides in solid solutions, our computations show that close to the absolute zero Cu(ii)–N_6_ complexes can undoubtedly rearrange exclusively through QMT from the ground state.

**Fig. 3:**
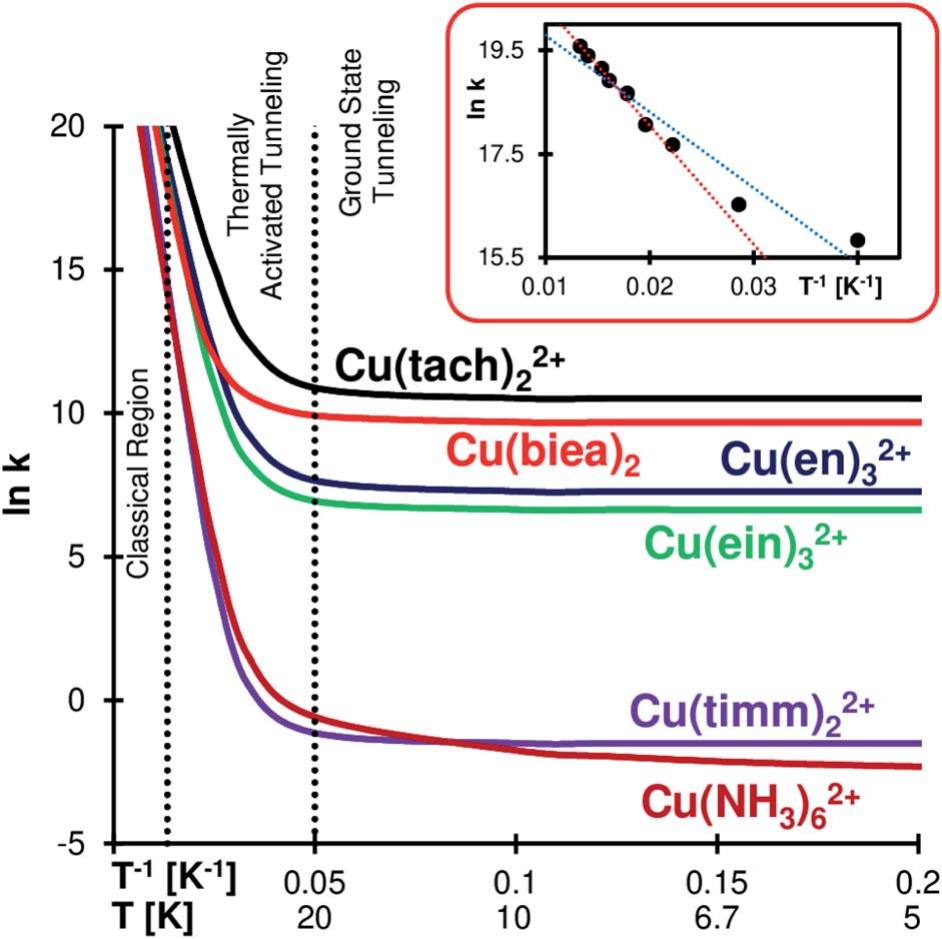
Arrhenius plots for the gas phase Cu(ii)–N_6_ complexes. Dotted vertical lines separate the regions according to their reactivity mechanism (classical region: negligible QMT; ground state tunnelling: QMT mostly from the lower vibrational mode; thermally activated tunnelling: the occupation of higher vibrational states enhances the rate by making the barrier lower and narrower).^66,67^ Inset: Cu(en)_3_SO_4_ solid state experimental results obtained by Bertini *et al.*;^34^ the dotted blue line is the original fitting, while the red line is the trend obtained from the six highest temperature points, where we can see the transition from the classical to the thermally activated regime.

Rate constant ratios for reactions over and through the barrier can be as high as 100 orders of magnitude at liquid He temperatures (see Cu(timm)_2_^2+^). The QMT effect at low temperature is evident in the Arrhenius plots of Fig. 3 (see full tables in the ESI†). The most striking case is Cu(biea)_2_, with a rate constant of 2 × 10^4^ s^−1^ (half-life of 34 μs). With this pincer ligand the geometry is highly constrained, lowering the *R*_JT_ and enhancing the QMT (Cu(tach)_2_^2+^ produces slightly faster tunnelling helped by the significantly lower barrier).

In all the studied systems SCT shows a negligible tunnelling contribution at *T* ≳ 75 K. This qualitatively matches the calculated crossover temperatures (see the ESI†)^62–65^ and explains why there is a negligible QMT in solid state Cu^II^–N_6_ complexes at liquid N_2_ and higher temperatures^37–40^ (although there is a considerable difference between these studies and our gas phase computations). Below ∼75 K there is a growing influence of thermally activated tunnelling (that is, QMT from vibrationally excited states). For most systems, below ∼20 K the reaction is exclusively driven by tunnelling from the ground state.

However, Cu(NH_3_)_6_^2+^ still shows signs of thermally activated tunnelling at an extremely low value of ∼4 K. We believe that this is caused by the almost free rotation of the amines, creating a virtually continuous band of vibrational states (in reality, hindered-rotational states) which enables the occupation of excited states even close to the absolute zero. This generates an interesting KIE profile, as we shall see below.

For comparison, Cu(H_2_O)_6_^2+^, which as explained before must have an enormous probability of tunnelling due to its low barrier (Δ*E*^‡^ = 1.3 kJ mol^−1^, with *R*_JT_ = 0.31 Å), has computed gas phase rates of *k*_SCT_ = 2 × 10^10^ s^−1^ and *k*_CVT_ = 5 × 10^−8^ s^−1^ at 4 K; this explains why the QMT was, supposedly, never observed for N-ligands, but easily seen in oxides. Still, regardless of the slower rates, all our species react well within what can be considered as “laboratory observable time”. They are too slow to be observed by cryogenic EPR (a method with timescales of the order of nanoseconds), but they might be observable by peak coalescence of exchanging atoms in cryogenic solid-state NMR,^68^ which has a much slower timescale. For instance, using the methodology described elsewhere,^16,17,61^ with a 500 MHz equipment (50.6 MHz for N) and with computed Δ*δ* values of 28, 35 and 2.9 for the central nitrogens, lateral nitrogens and lateral hydrogens of Cu(biea)_2_^2+^, respectively, we obtain coalescence rate constants of 3000–4000 s^−1^. In this situation, we predict merged NMR peaks for this complex at any temperature, while in the absence of QMT we would see two peaks below ∼30 K.

Yet, the QMT rearrangement on one Cu–N_6_ complex might have been detected by EPR four decades ago, even if the authors of the study did not realize it (they were probably not aware of the QMT mechanism, as the idea was in its infancy). The rate of the solid-state dynamic^69^ JT automerization of Cu(en)_3_SO_4_ was measured by Bertini *et al.*^34^ from the temperature dependence of the EPR line width and Hudson's equation.^70^ The obtained Arrhenius plot was found to be acceptably linear, from which the activation energy was calculated (*E*_a_ = 1.22 kJ mol^−1^). We redraw here their original data on a different scale (inset in Fig. 3, see also Table S7 in the ESI†), which highlights the concavity of the plot, a signature of thermally activated QMT. If we consider only the first six points of the graph (the ones with apparent linearity), we can see that the steepest slope produces a higher *E*_a_ of 1.91 kJ mol^−1^ (our computed gas phase value is much higher – *E*_a_ = 6.1 kJ mol^−1^ – depicting the differences between the methods and conditions).

Due to the computational cost to obtain accurate SCT computations, we could not test larger ligands. However, it is possible to artificially set heavier atoms in simple complexes to model the ligand size effect. For that, we studied the Cu(NH_3_)_6_^2+^ system changing the hydrogen masses from 1 to 2, 4, 8 and 16 u, equivalent to ligands' masses of 17, 20, 26, 38 and 62 u per NH_3_ (for comparison, imidazole, the ligand originally supposed to tunnel in solid state,^36–40^ has a mass of 68 u). The results are clear: at 4 K the rates are 9 × 10^−2^, 8 × 10^−3^, 4 × 10^−5^, 5 × 10^−9^ and 4 × 10^−15^ s^−1^, showing the difficulties of tunnelling if large ligands are attached (see Table S6 in the ESI†). In the case of imidazoles, it is still possible that some QMT can occur in solid state due to the crystal constraints, but in the gas phase it would be completely impossible. It is worth mentioning that with chelating ligands, like most of our cases, the effective moving mass is relatively light, as many atoms in the framework have almost negligible movement.

**Fig. 4:**
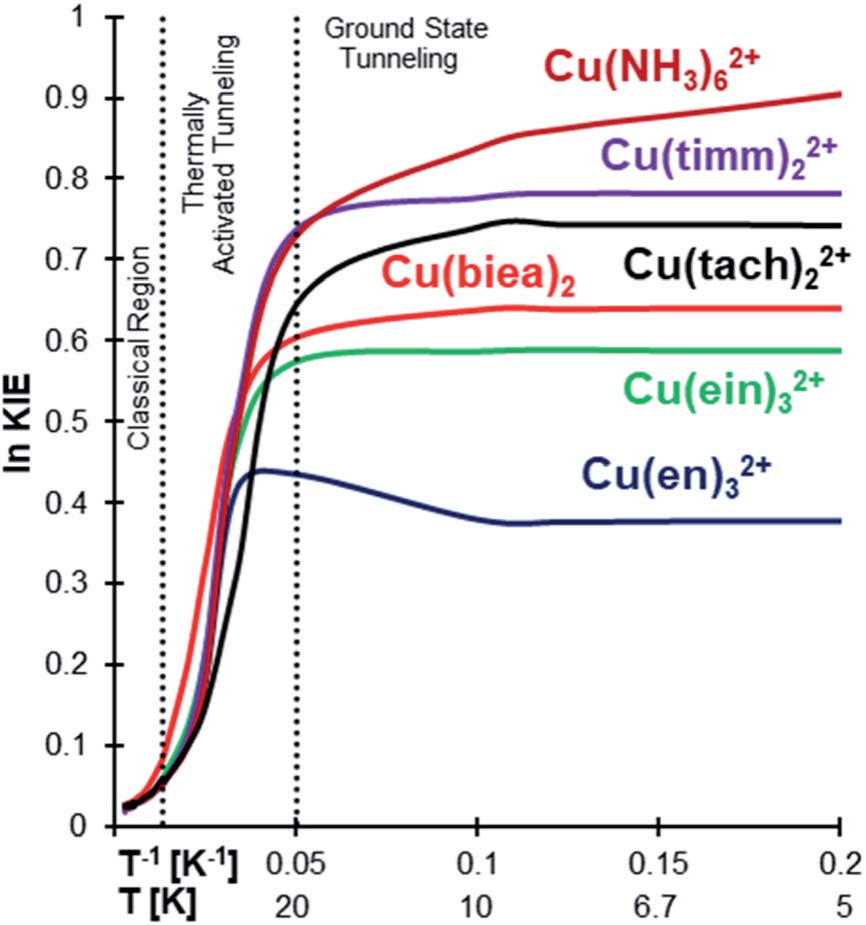
ln KIE *vs. T*^−1^ for Cu(ii)–N_6_ type complexes in the gas phase for ^14^N/^15^N substitution in all the nitrogens. All the complexes are showing a large KIE at low temperatures.

KIE analyses were carried out by replacing all ^14^N isotopes with their heavier ^15^N to assess the outcome of a possible experimental test. As can be seen in Fig. 4 (also Tables 1 and S8 in the ESI†), the high KIE plateau at low temperature clearly indicates ground state tunnelling.^11,71^

The exception is Cu(NH_3_)_6_^2+^ which, as previously discussed, does not easily converge into the temperature independence range. The ^14^N/^15^N KIE grows first due to conventional ZPE differences and then in a much stepper way due to thermally activated tunnelling. However, below ∼10 K instead of stabilizing in a plateau, like all the other systems, it continues growing (Fig. 4 and S1 in the ESI†), due to the almost free rotation of the ammonia groups, as explained above.

Considering this, we also calculated the H/D KIE (all hydrogens substituted), as the higher mass of the deuterium should hinder the free NH_3_ rotation. The results were unanticipated and possibly unique, as the H/D KIE decreases below 10 K (Fig. S1 in the ESI†). This strange behaviour is caused by the ND_3_ system converging to QMT from the ground state at higher temperatures compared to NH_3_. Noteworthily, computing accurate properties from such flat potentials (especially conformational surfaces that are taken as vibrations) is problematic, and therefore we can only take this observation as a hypothesis more than a real prediction.

## Conclusions

Significant heavy atom tunnelling was theoretically proven to occur in the gas-phase degenerate rearrangement of Jahn–Teller distorted Cu(ii)–N_6_ complexes. While similar QMT has been experimentally observed in Cu^2+^ oxide solids due to their much lower barriers, the evidence in common nitrogen-based complexes was found to be more elusive. Herein we show that easily synthesizable mono-, bi- and tri-dentate amine and imine ligands can react by tunnelling under cryogenic conditions, although at rates that are hard to detect by standard experimental tests. Surprisingly (and unbeknown to the authors), a solid state EPR experimental manifestation of our theoretical gas phase results apparently emerged 40 years ago.

Nitrogen KIE analysis on all the tested complexes revealed a large KIE, with an unexpected behaviour of Cu(NH_3_)_6_^2+^, apparently due to free rotation of the ammonia groups. We plan to synthesize some of the Cu(ii)–N_6_ complexes to test them by EPR characterization.

## Conflicts of interest

There are no conflicts to declare.

## Supplementary Material

SC-011-D0SC00160K-s001
