# Supplementary material for: Heavy-atom tunnelling in Cu(ii)N6 complexes: theoretical predictions and experimental manifestation
Source: Chem Sci. 2020 Feb 18;11(10):2828–33. doi: 10.1039/d0sc00160k (PMC8157485; doi:10.1039/d0sc00160k)
Supplement: SC-011-D0SC00160K-s001 [file SC-011-D0SC00160K-s001.pdf]

Electronic Supplementary Material (ESI) for Chemical Science.
This journal is © The Royal Society of Chemistry 2020

Heavy-Atom Tunnelling in Cu(II)N_6 Complexes: Theoretical Predictions and Experimental Manifestation

Supporting Information

Itzhak Sedgi,[†,‡] and Sebastian Kozuch,*[,†]
[†] Department of Chemistry, Ben-Gurion University of the Negev, Beer-Sheva 841051, Israel.
[‡] Department of Analytical Chemistry, Nuclear Research Center Negev. P.O Box 9001, Beer-Sheva, Israel.

Table of Contents

Energy Benchmark	S2
Rate Constants	S4
Rate constants for solid $Cu(en)_3SO_4$	S11
KIE	S12
Crossover temperatures	S13
Example of Polyrate input file	S14
XYZ optimized geometries	S15
References	S19

Energy Benchmark

DLPNO-CCSD(T)/aug-cc-pvqz//MN15/Def2-TZVPD was used for benchmark reference activation energies in the degenerate rearrangement of $Cu(en)_3^{+2}$, $Cu(ein)_3^{+2}$, $Cu(NH_3)_6^{+2}$, $Cu(timm)_2^{+2}$ and $Cu(biea)_2$. For $Cu(NH_3)_6^{+2}$ we tested several PNO schemes (loose, normal, tight and an “extra-tight” with home-made stricter parameters). While the first two had some variability, the difference between the tightPNO and our stricter values was only 0.3 kJ·mol^{-1}, indicating convergence in this front. Therefore, we computed all the DLPNO-CCSD(T) values with tightPNO criteria.

T1 and %TAE(T) diagnostics were carried out to test the viability of coupled-cluster single reference benchmarks. In all cases (Table S4) the results indicate very mild nondynamical correlation (T1 << 0.05 for first row transition metals,[1] and %TAE(T) << 5%.[2]

DLPNO-CCSD(T) computations were carried out with ORCA4.0, while DFT results were obtained with Gaussian16. We selected a set of seven functionals known for their relatively good performance on barriers in complexes and/or their inexpensiveness. Similarly, five small basis sets were considered. From these results PBE0/6-31+G(d) was selected as the best compromise (Tables S1 and S2).

Table S1. Mean error (kJ·mol^{-1}) for the five complexes.

	TPSSh	MN15	MN15L	ωB97X	PBE0	PBE	B97D3
Def2-SV	2.2	2.6	4.6	1.8	2.5	2.3	2.5
Def2-SVP	2.3	3.1	4.7	1.9	2.7	2.3	2.4
Def2-TZVP	0.4	0.9	1.5	0.5	0.6	1.0	1.8
6-31G(d)	2.8	1.1	1.6	2.2	1.1	5.2	3.7
6-31+G(d)	0.6	2.2	3.0	1.1	0.4	1.1	2.0

Table S2. Maximum error (kJ·mol^{-1}) for the five complexes.

	TPSSh	MN15	MN15L	ωB97X	PBE0	PBE	B97D3
Def2-SV	3.2	3.8	6.5	2.5	3.6	3.7	4.8
Def2-SVP	3.3	3.7	6.3	2.9	3.7	3.6	4.8
Def2-TZVP	1.0	1.9	3.2	1.1	1.3	1.9	3.0
6-31G(d)	4.4	2.8	4.1	3.8	3.2	7.9	6.5
6-31+G(d)	1.3	3.9	4.2	2.1	0.9	1.7	2.4

Table S3. $\Delta E^{\ddagger}$ (kJ·mol^{-1}) for the degenerate rearrangement of Cu(II) with the depicted ligands.

Ligand		ein	timm	en	NH_3	biea
TPSSh	Def2-SV	5.20	6.53	3.75	4.99	7.05
	Def2-SVP	4.98	6.36	3.85	4.85	6.41
	Def2-TZVP	7.21	9.52	5.99	8.09	7.41
	6-31G(d)	8.77	11.76	8.63	11.49	11.32
	6-31+G(d)	7.85	9.60	6.88	8.64	8.19
MN15	Def2-SV	4.78	6.31	3.15	4.65	7.51
	Def2-SVP	4.61	6.19	3.30	4.69	0.00
	Def2-TZVP	6.52	8.50	5.11	7.32	7.64
	6-31G(d)	7.53	9.53	5.20	8.03	9.66
	6-31+G(d)	5.07	5.18	4.01	6.14	6.75
MN15L	Def2-SV	3.69	5.34	0.44	1.97	3.89
	Def2-SVP	3.49	5.25	0.68	1.97	3.26
	Def2-TZVP	6.29	9.11	3.74	6.03	5.38
	6-31G(d)	6.34	8.92	2.87	5.10	6.88
	6-31+G(d)	4.98	6.03	2.80	4.56	4.65
ωB97X	Def2-SV	5.23	7.06	4.44	0.00	7.54
	Def2-SVP	5.03	6.88	4.49	5.20	6.84
	Def2-TZVP	7.18	10.17	6.64	8.25	7.85
	6-31G(d)	0.00	11.42	7.48	0.00	10.69
	6-31+G(d)	7.89	10.40	7.57	8.74	8.97
PBE0	Def2-SV	4.67	6.24	3.53	4.56	6.77
	Def2-SVP	4.46	6.06	3.63	4.48	6.07
	Def2-TZVP	6.51	9.22	5.62	7.44	6.81
	6-31G(d)	8.22	8.92	6.83	8.90	10.06
	6-31+G(d)	7.30	9.39	6.43	8.05	7.83
PBE	Def2-SV	4.98	6.85	3.29	5.19	7.18
	Def2-SVP	5.28	6.60	3.33	4.78	6.46
	Def2-TZVP	7.45	10.97	5.40	7.51	7.47
	6-31G(d)	8.90	14.11	11.95	16.06	12.96
	6-31+G(d)	8.45	10.66	6.73	8.93	8.62
B97D3	Def2-SV	5.73	7.78	2.14	3.91	7.50
	Def2-SVP	5.99	7.58	2.15	3.57	6.92
	Def2-TZVP	7.94	10.97	3.97	6.06	8.11
	6-31G(d)	11.59	15.53	7.85	11.07	0.00
	6-31+G(d)	8.95	11.06	4.92	6.56	9.26
Ref.		7.13	9.06	6.97	8.14	6.89

Table S4. Static correlation diagnostics.

	T1		%TAE(T)	
Ligand	React.	TS	React.	TS
biea	0.019	0.018	2.5	2.5
ein	0.017	0.016	2.4	2.4
en	0.014	0.014	1.5	1.5
timm	0.017	0.016	2.3	2.3
NH3	0.015	0.014	1.4	1.4

Table S5. CVT and CVT+SCT rate constants (in s^{-1}). QRST not included over ~50 K.

$Cu(NH_3)_6^{2+}$

T(K)	CVT	CVT+SCT
4	9.04E-88	8.61E-02
6	5.13E-55	1.10E-01
8	1.24E-38	1.40E-01
10	8.52E-29	1.74E-01
20	4.21E-09	5.60E-01
30	1.57E-02	4.77E+00
40	2.98E+01	2.33E+02
50	2.72E+03	8.23E+03
75	1.08E+06	1.66E+06
77.36	1.55E+06	2.32E+06
100	2.11E+07	2.67E+07
125	1.26E+08	1.45E+08
150	4.12E+08	4.56E+08
175	9.65E+08	1.04E+09
194.7	1.62E+09	1.72E+09
200	1.83E+09	1.94E+09
225	3.01E+09	3.15E+09
250	4.49E+09	4.66E+09
273.15	6.10E+09	6.29E+09
275	6.24E+09	6.43E+09
298.15	8.06E+09	8.27E+09
300	8.21E+09	8.42E+09
325	1.04E+10	1.06E+10
350	1.27E+10	1.29E+10
373.15	1.49E+10	1.51E+10
375	1.51E+10	1.53E+10
400	1.76E+10	1.78E+10

$Cu(NH_3)_6^{2+}$ [^{15}N]

T(K)	CVT	CVT+SCT
4	6.80E-88	3.39E-02
6	4.24E-55	4.54E-02
8	1.08E-38	5.91E-02
10	7.59E-29	7.53E-02
20	3.96E-09	2.70E-01
30	1.50E-02	2.94E+00
40	2.87E+01	1.94E+02
50	2.62E+03	7.44E+03
75	1.04E+06	1.57E+06
77.36	1.50E+06	2.20E+06
100	2.05E+07	2.56E+07
125	1.22E+08	1.40E+08
150	4.00E+08	4.41E+08
175	9.38E+08	1.01E+09
194.7	1.57E+09	1.67E+09
200	1.78E+09	1.88E+09
225	2.93E+09	3.05E+09
250	4.37E+09	4.52E+09
273.15	5.94E+09	6.11E+09
275	6.07E+09	6.25E+09
298.15	7.84E+09	8.03E+09
300	7.99E+09	8.18E+09
325	1.01E+10	1.03E+10
350	1.23E+10	1.26E+10
373.15	1.45E+10	1.47E+10
375	1.47E+10	1.49E+10
400	1.71E+10	1.73E+10

$Cu(en)_3^{2+}$

T(K)	CVT	CVT+SCT
4	4.65E-63	1.45E+03
6	1.81E-38	1.45E+03
8	3.90E-26	1.45E+03
10	1.03E-18	1.46E+03
20	9.02E-04	2.12E+03
30	9.78E+01	1.08E+04
40	3.35E+04	1.91E+05
50	1.13E+06	3.14E+06
75	1.25E+08	1.87E+08
77.36	1.66E+08	2.43E+08
100	1.33E+09	1.66E+09
125	5.52E+09	6.35E+09
150	1.43E+10	1.58E+10
175	2.84E+10	3.05E+10
194.7	4.30E+10	4.56E+10
200	4.75E+10	5.01E+10
225	7.09E+10	7.40E+10
250	9.79E+10	1.01E+11
273.15	1.25E+11	1.29E+11
275	1.28E+11	1.31E+11
298.15	1.57E+11	1.60E+11
300	1.59E+11	1.63E+11
325	1.92E+11	1.96E+11
350	2.25E+11	2.29E+11
373.15	2.57E+11	2.61E+11
375	2.59E+11	2.63E+11
400	2.93E+11	2.97E+11

$Cu(en)_3^{2+}$ [^{15}N]

T(K)	CVT	CVT+SCT
4	3.03E-63	9.93E+02
6	1.36E-38	9.93E+02
8	3.15E-26	9.94E+02
10	8.68E-19	9.98E+02
20	8.27E-04	1.37E+03
30	9.21E+01	6.59E+03
40	3.19E+04	1.79E+05
50	1.08E+06	2.83E+06
75	1.21E+08	1.77E+08
77.36	1.61E+08	2.31E+08
100	1.29E+09	1.59E+09
125	5.37E+09	6.13E+09
150	1.40E+10	1.53E+10
175	2.77E+10	2.96E+10
194.7	4.20E+10	4.43E+10
200	4.63E+10	4.87E+10
225	6.92E+10	7.21E+10
250	9.55E+10	9.87E+10
273.15	1.22E+11	1.26E+11
275	1.24E+11	1.28E+11
298.15	1.53E+11	1.56E+11
300	1.55E+11	1.59E+11
325	1.87E+11	1.91E+11
350	2.20E+11	2.24E+11
373.15	2.51E+11	2.54E+11
375	2.53E+11	2.57E+11
400	2.86E+11	2.90E+11

$Cu(ein)_3^{2+}$

T(K)	CVT	CVT+SCT
4	1.76E-71	7.69E+02
6	4.42E-44	7.69E+02
8	2.41E-30	7.70E+02
10	4.42E-22	7.72E+02
20	1.90E-05	1.04E+03
30	7.78E+00	3.98E+03
40	5.31E+03	6.09E+04
50	2.75E+05	1.01E+06
75	5.53E+07	9.12E+07
77.36	7.66E+07	1.22E+08
100	8.04E+08	1.05E+09
125	4.05E+09	4.80E+09
150	1.20E+10	1.35E+10
175	2.61E+10	2.84E+10
194.7	4.20E+10	4.50E+10
200	4.70E+10	5.01E+10
225	7.43E+10	7.82E+10
250	1.07E+11	1.12E+11
273.15	1.42E+11	1.47E+11
275	1.45E+11	1.50E+11
298.15	1.84E+11	1.89E+11
300	1.87E+11	1.92E+11
325	2.31E+11	2.37E+11
350	2.78E+11	2.84E+11
373.15	3.23E+11	3.29E+11
375	3.26E+11	3.32E+11
400	3.75E+11	3.81E+11

$Cu(ein)_3^{2+}$ [^{15}N]

T(K)	CVT	CVT+SCT
4	1.06E-71	4.27E+02
6	3.14E-44	4.27E+02
8	1.86E-30	4.27E+02
10	3.60E-22	4.29E+02
20	1.71E-05	5.85E+02
30	7.26E+00	2.50E+03
40	5.04E+03	4.88E+04
50	2.63E+05	8.91E+05
75	5.34E+07	8.57E+07
77.36	7.39E+07	1.15E+08
100	7.79E+08	1.01E+09
125	3.94E+09	4.62E+09
150	1.17E+10	1.30E+10
175	2.54E+10	2.76E+10
194.7	4.09E+10	4.37E+10
200	4.58E+10	4.87E+10
225	7.24E+10	7.60E+10
250	1.05E+11	1.09E+11
273.15	1.39E+11	1.43E+11
275	1.42E+11	1.46E+11
298.15	1.79E+11	1.84E+11
300	1.82E+11	1.87E+11
325	2.26E+11	2.31E+11
350	2.71E+11	2.77E+11
373.15	3.15E+11	3.20E+11
375	3.18E+11	3.24E+11
400	3.66E+11	3.72E+11

$Cu(timm)_2^{2+}$

T(K)	CVT	CVT+SCT
4	1.90E-101	2.23E-01
6	4.64E-64	2.23E-01
8	2.50E-45	2.23E-01
10	4.54E-34	2.24E-01
20	1.91E-11	3.20E-01
30	7.73E-04	1.75E+00
40	5.16E+00	7.96E+01
50	1.03E+03	4.19E+03
75	1.23E+06	2.08E+06
77.36	1.89E+06	3.09E+06
100	4.23E+07	5.61E+07
125	3.55E+08	4.24E+08
150	1.47E+09	1.66E+09
175	4.06E+09	4.44E+09
194.7	7.54E+09	8.10E+09
200	8.72E+09	9.33E+09
225	1.58E+10	1.67E+10
250	2.55E+10	2.66E+10
273.15	3.67E+10	3.81E+10
275	3.77E+10	3.91E+10
298.15	5.11E+10	5.27E+10
300	5.23E+10	5.39E+10
325	6.90E+10	7.08E+10
350	8.75E+10	8.94E+10
373.15	1.06E+11	1.08E+11
375	1.08E+11	1.10E+11
400	1.29E+11	1.31E+11

$Cu(timm)_2^{2+}$ [^{15}N]

T(K)	CVT	CVT+SCT
4	1.33E-101	1.02E-01
6	3.65E-64	1.02E-01
8	2.09E-45	1.02E-01
10	3.94E-34	1.03E-01
20	1.78E-11	1.53E-01
30	7.35E-04	1.05E+00
40	4.96E+00	6.37E+01
50	9.98E+02	3.74E+03
75	1.19E+06	1.97E+06
77.36	1.83E+06	2.93E+06
100	4.11E+07	5.39E+07
125	3.46E+08	4.10E+08
150	1.43E+09	1.61E+09
175	3.96E+09	4.31E+09
194.7	7.36E+09	7.88E+09
200	8.51E+09	9.08E+09
225	1.54E+10	1.63E+10
250	2.49E+10	2.60E+10
273.15	3.59E+10	3.72E+10
275	3.69E+10	3.81E+10
298.15	5.00E+10	5.15E+10
300	5.11E+10	5.26E+10
325	6.74E+10	6.91E+10
350	8.55E+10	8.73E+10
373.15	1.04E+11	1.06E+11
375	1.05E+11	1.07E+11
400	1.26E+11	1.28E+11

Cu(biea)$_2$

T(K)	CVT	CVT+SCT
4	2.95E-75	1.60E+04
6	1.34E-46	1.60E+04
8	3.12E-32	1.60E+04
10	1.36E-23	1.61E+04
20	3.26E-06	2.03E+04
30	2.30E+00	4.11E+04
40	2.04E+03	1.61E+05
50	1.23E+05	1.12E+06
75	3.00E+07	6.54E+07
77.36	4.20E+07	8.67E+07
100	4.82E+08	7.26E+08
125	2.59E+09	3.34E+09
150	8.03E+09	9.55E+09
175	1.81E+10	2.06E+10
194.7	2.98E+10	3.30E+10
200	3.35E+10	3.69E+10
225	5.42E+10	5.85E+10
250	7.98E+10	8.48E+10
273.15	1.07E+11	1.13E+11
275	1.10E+11	1.15E+11
298.15	1.40E+11	1.47E+11
300	1.43E+11	1.49E+11
325	1.79E+11	1.86E+11
350	2.18E+11	2.25E+11
373.15	2.55E+11	2.62E+11
375	2.58E+11	2.65E+11
400	2.99E+11	3.06E+11

Cu(biea)$_2$ [^{15}N]

T(K)	CVT	CVT+SCT
4	1.52E-75	8.44E+03
6	8.62E-47	8.44E+03
8	2.24E-32	8.45E+03
10	1.05E-23	8.51E+03
20	2.85E-06	1.11E+04
30	2.11E+00	2.47E+04
40	1.91E+03	1.16E+05
50	1.16E+05	9.15E+05
75	2.88E+07	5.99E+07
77.36	4.04E+07	7.99E+07
100	4.66E+08	6.86E+08
125	2.51E+09	3.20E+09
150	7.80E+09	9.20E+09
175	1.76E+10	1.99E+10
194.7	2.90E+10	3.20E+10
200	3.26E+10	3.58E+10
225	5.28E+10	5.68E+10
250	7.78E+10	8.25E+10
273.15	1.05E+11	1.10E+11
275	1.07E+11	1.12E+11
298.15	1.37E+11	1.43E+11
300	1.40E+11	1.45E+11
325	1.75E+11	1.81E+11
350	2.13E+11	2.19E+11
373.15	2.49E+11	2.55E+11
375	2.52E+11	2.58E+11
400	2.92E+11	2.99E+11

$Cu(tach)_2^{2+}$

T(K)	CVT	CVT+SCT
4	4.27E-50	3.69E+04
6	7.99E-30	3.70E+04
8	1.19E-19	3.70E+04
10	1.59E-13	3.71E+04
20	3.60E-01	5.34E+04
30	5.51E+03	2.48E+05
40	7.27E+05	3.78E+06
50	1.40E+07	3.59E+07
75	7.61E+08	1.11E+09
77.36	9.72E+08	1.39E+09
100	5.76E+09	7.08E+09
125	1.96E+10	2.24E+10
150	4.47E+10	4.89E+10
175	8.08E+10	8.63E+10
194.7	1.16E+11	1.22E+11
200	1.26E+11	1.33E+11
225	1.79E+11	1.86E+11
250	2.37E+11	2.44E+11
273.15	2.93E+11	3.01E+11
275	2.98E+11	3.06E+11
298.15	3.56E+11	3.65E+11
300	3.61E+11	3.69E+11
325	4.25E+11	4.33E+11
350	4.89E+11	4.97E+11
373.15	5.48E+11	5.56E+11
375	5.53E+11	5.61E+11
400	6.15E+11	6.23E+11

$Cu(tach)_2^{2+}$ [^{15}N]

T(K)	CVT	CVT+SCT
4	2.34E-50	1.76E+04
6	5.35E-30	1.76E+04
8	8.81E-20	1.76E+04
10	1.25E-13	1.77E+04
20	3.19E-01	2.80E+04
30	5.08E+03	1.82E+05
40	6.83E+05	3.25E+06
50	1.33E+07	3.25E+07
75	7.32E+08	1.05E+09
77.36	9.36E+08	1.31E+09
100	5.57E+09	6.79E+09
125	1.90E+10	2.16E+10
150	4.35E+10	4.74E+10
175	7.87E+10	8.38E+10
194.7	1.13E+11	1.19E+11
200	1.23E+11	1.29E+11
225	1.74E+11	1.81E+11
250	2.31E+11	2.38E+11
273.15	2.86E+11	2.93E+11
275	2.91E+11	2.98E+11
298.15	3.48E+11	3.55E+11
300	3.52E+11	3.60E+11
325	4.15E+11	4.22E+11
350	4.77E+11	4.85E+11
373.15	5.35E+11	5.42E+11
375	5.39E+11	5.47E+11
400	6.00E+11	6.07E+11

Table S6. Reaction rate constants [s^{-1}] of $Cu(NH_3)_6^{+2}$, for different hydrogen masses.

	2H		4H		8H		16H	
T(K)	CVT	CVT+SCT	CVT	CVT+SCT	CVT	CVT+SCT	CVT	CVT+SCT
4	6.65E-89	7.66E-03	8.52E-90	4.45E-05	1.71E-90	5.20E-09	4.95E-91	3.87E-15
6	8.32E-56	9.19E-03	1.93E-56	5.36E-05	6.08E-57	6.52E-09	2.46E-57	5.11E-15
8	3.00E-39	1.11E-02	9.42E-40	6.58E-05	3.73E-40	8.77E-09	1.80E-40	8.02E-15
10	2.61E-29	1.34E-02	9.87E-30	8.25E-05	4.51E-30	1.26E-08	2.44E-30	1.59E-14
20	2.11E-09	4.64E-02	1.18E-09	5.27E-04	7.27E-10	7.78E-07	4.86E-10	5.56E-09
30	9.33E-03	8.47E-01	5.95E-03	9.70E-02	4.02E-03	1.70E-02	2.81E-03	5.93E-03
40	1.94E+01	9.99E+01	1.32E+01	4.10E+01	9.27E+00	1.80E+01	6.58E+00	9.56E+00
50	1.87E+03	4.48E+03	1.33E+03	2.51E+03	9.50E+02	1.39E+03	6.79E+02	8.49E+02
75	8.05E+05	1.10E+06	6.02E+05	7.68E+05	4.44E+05	5.13E+05	3.22E+05	3.52E+05
77.36	1.16E+06	1.55E+06	8.72E+05	1.09E+06	6.45E+05	7.37E+05	4.69E+05	5.09E+05
100	1.65E+07	1.92E+07	1.27E+07	1.44E+07	9.54E+06	1.02E+07	7.03E+06	7.35E+06
125	1.01E+08	1.10E+08	7.89E+07	8.50E+07	6.03E+07	6.27E+07	4.49E+07	4.60E+07
150	3.37E+08	3.55E+08	2.68E+08	2.81E+08	2.07E+08	2.12E+08	1.55E+08	1.58E+08
175	7.99E+08	8.27E+08	6.45E+08	6.66E+08	5.02E+08	5.09E+08	3.78E+08	3.82E+08
194.7	1.35E+09	1.39E+09	1.10E+09	1.13E+09	8.60E+08	8.69E+08	6.49E+08	6.54E+08
200	1.53E+09	1.57E+09	1.25E+09	1.28E+09	9.76E+08	9.85E+08	7.37E+08	7.42E+08
225	2.55E+09	2.59E+09	2.09E+09	2.12E+09	1.64E+09	1.65E+09	1.24E+09	1.25E+09
250	3.83E+09	3.87E+09	3.16E+09	3.20E+09	2.49E+09	2.50E+09	1.89E+09	1.89E+09
273.15	5.24E+09	5.27E+09	4.33E+09	4.37E+09	3.42E+09	3.43E+09	2.60E+09	2.60E+09
275	5.36E+09	5.39E+09	4.44E+09	4.48E+09	3.50E+09	3.51E+09	2.66E+09	2.66E+09
298.15	6.96E+09	6.99E+09	5.78E+09	5.82E+09	4.57E+09	4.57E+09	3.47E+09	3.47E+09
300	7.10E+09	7.12E+09	5.89E+09	5.93E+09	4.66E+09	4.66E+09	3.54E+09	3.54E+09
325	9.00E+09	9.02E+09	7.49E+09	7.53E+09	5.94E+09	5.94E+09	4.51E+09	4.51E+09
350	1.10E+10	1.10E+10	9.21E+09	9.25E+09	7.31E+09	7.30E+09	5.55E+09	5.55E+09
373.15	1.30E+10	1.30E+10	1.09E+10	1.09E+10	8.64E+09	8.63E+09	6.57E+09	6.57E+09
375	1.32E+10	1.32E+10	1.10E+10	1.11E+10	8.75E+09	8.74E+09	6.65E+09	6.65E+09
400	1.54E+10	1.54E+10	1.29E+10	1.29E+10	1.02E+10	1.02E+10	7.79E+09	7.79E+09

Table S7. Experimental automerization rate constants for solid state $Cu(en)_3SO_4$.[3]

T(K)	k [s^{-1}]	ln(k)
25	7.50E+06	15.83
35	1.50E+07	16.52
45	4.75E+07	17.68
51	7.00E+07	18.06
56	1.28E+08	18.66
62	1.63E+08	18.91
65	2.06E+08	19.14
71	2.63E+08	19.39
75	3.16E+08	19.57

Table S8. Kinetic isotope effect from CVT+SCT rate constants

T(K)	$Cu(NH_3)_6^{+2}$	$Cu(en)_3^{+2}$	$Cu(ein)_3^{+2}$	$Cu(timm)_2^{+2}$	$Cu(tach)_2^{+2}$	$Cu(biea)_2$
4	2.54	1.46	1.80	2.19	2.10	1.90
6	2.42	1.46	1.80	2.19	2.10	1.90
8	2.37	1.46	1.80	2.19	2.10	1.89
10	2.31	1.46	1.80	2.17	2.10	1.89
20	2.07	1.55	1.78	2.09	1.91	1.83
30	1.62	1.52	1.59	1.67	1.36	1.66
40	1.20	1.20	1.25	1.25	1.16	1.39
50	1.11	1.11	1.13	1.12	1.10	1.22
75	1.06	1.06	1.06	1.06	1.06	1.09
77.36	1.05	1.05	1.06	1.05	1.06	1.09
100	1.04	1.04	1.04	1.04	1.04	1.06
125	1.04	1.04	1.04	1.03	1.04	1.04
150	1.03	1.03	1.04	1.03	1.03	1.04
175	1.03	1.03	1.03	1.03	1.03	1.04
194.7	1.03	1.03	1.03	1.03	1.03	1.03
200	1.03	1.03	1.03	1.03	1.03	1.03
225	1.03	1.03	1.03	1.02	1.03	1.03
250	1.03	1.02	1.03	1.02	1.03	1.03
273.15	1.03	1.02	1.03	1.02	1.03	1.03
275	1.03	1.02	1.03	1.03	1.03	1.03
298.15	1.03	1.03	1.03	1.02	1.03	1.03
300	1.03	1.03	1.03	1.02	1.03	1.03
325	1.03	1.03	1.03	1.02	1.03	1.03
350	1.02	1.02	1.03	1.02	1.02	1.03
373.15	1.03	1.03	1.03	1.02	1.03	1.03
375	1.03	1.02	1.02	1.03	1.03	1.03
400	1.03	1.02	1.02	1.02	1.03	1.02

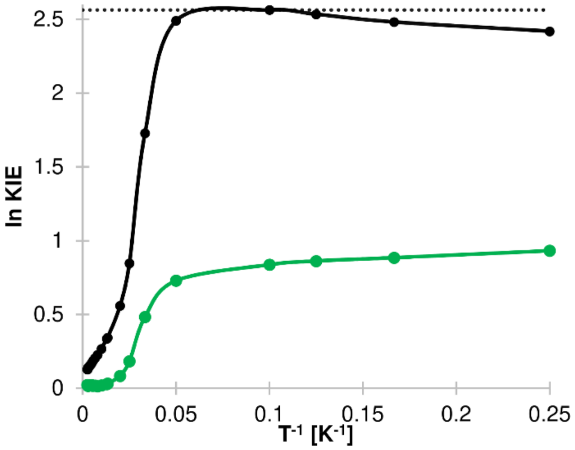

Figure S1. ln KIE vs T^{-1} for H/D (black) and $^{14}N/^{15}N$ (green) KIEs of $Cu(NH_3)_2^{2+}$. Dotted line, as a reference, shows the maximum KIE (at 10 K).

Table S9. Crossover temperatures.

	$\tilde{\nu}$ (cm^{-1})	$\tilde{\omega}$ (rad cm^{-1})	ω (s^{-1})	T_C
$Cu(biea)_2$	208.3	1308	3.93E+13	47.7
$Cu(ein)_3^{+2}$	172.4	1083	3.25E+13	39.5
$Cu(en)_3^{+2}$	159.4	1002	3.01E+13	36.5
$Cu(timm)_2^{+2}$	176.1	1106	3.32E+13	40.3
$Cu(NH3)_6^{+2}$	161.8	1017	3.05E+13	37.1
$Cu(tach)_2^{+2}$	154.6	972	2.91E+13	35.4

The crossover temperature is measured as $T_C = \hbar\omega / 2\pi k_B$, where ω is the imaginary frequency at the transition state corresponding to the reaction coordinate.

Example of Polyrate input file (.dat file):

```
*GENERAL

TITLE

QMT_calculation_for_CuNH3_6
END

ATOMS
1 Cu
2 N
3 N
4 N
5 N
6 N
7 N
8 H
9 H
10 H
11 H
12 H
13 H
14 H
15 H
16 H
17 H
18 H
19 H
20 H
21 H
22 H
23 H
24 H
25 H
END

NOSUPERMOL

*SECOND

HESSCAL hhook

FPRINT

*OPTIMIZATION

PRINT

OPTMIN ohook
OPTTS ohook

*REACT1
INITGEO hooks
GEOM
1
2
3
4
5
6
7
8
9
10
11
12
13
14
15
16
17
18
19
20
21
22
23
24
25
END
SPECIES nonlinrp

*PROD1
INITGEO hooks
GEOM
1
2
3
4
5
6
7
8
9
10
11
12
13
14
15
16
17
18
19
20
21
22
23
24
25
END
SPECIES nonlinrp

*START
INITGEO hooks
GEOM
1
2
3
4
5
6
7
8
9
10
11
12
13
14
15
16
17
18
19
20
21
22
23
24
25
END
SPECIES nonlints
PROJECT

*PATH
SYMMETRY
INTMU 3
SSTEP 0.001
RPM pagem
SRANGE
  SLP 20.
  SLM -20.
END
SPECSTOP
  CURVE VMEP
  PERCENTDOWN 99
END
PRPATH
  coord 1 2
  xmol
  freq 69
END

*TUNNEL
ZCT
SCT
QRST
  harmonic
  mode 69
  states all
END

*RATE
FORWARDK
SIGMAF 1
TST
CVT
PRDELG
PRPART rtp

TEMP
4
6
8
10
20
30
40
50
75
77.355
100
125
150
175
194.7
200
225
250
273.15
275
298.15
300
325
350
373.15
375
400
END

ANALYSIS
4
6
8
10
20
30
40
50
75
77.355
100
125
150
175
194.7
200
225
250
273.15
275
298.15
300
325
350
373.15
375
400
END

EACT
6. 10.
10. 20.
20. 50.
50. 100.
200. 225.
300. 325.
END

GTLOG
```

XYZ optimized geometries at the PBE0/6-31+G(d) level.

$Cu(NH_3)_6^{2+}$

Reactant/Product

Cu	0.000000	0.000000	0.003747
N	-0.153290	1.450585	1.485711
N	2.555756	0.133947	-0.040956
N	0.000000	-1.483144	-1.450119
N	-2.555756	-0.133947	-0.040956
N	0.153290	-1.450585	1.485711
N	0.000000	1.483144	-1.450119
H	0.748775	1.730082	1.874370
H	3.030137	-0.608522	0.475194
H	-0.532558	-2.308327	-1.171846
H	-3.030137	0.608522	0.475194
H	-0.595826	2.316338	1.174718
H	2.953176	0.992008	0.344457
H	-0.415767	-1.195538	-2.336786
H	-2.960092	-0.079884	-0.977053
H	0.595826	-2.316338	1.174718
H	-0.935187	1.821442	-1.682518
H	0.532558	2.308327	-1.171846
H	-0.748775	-1.730082	1.874370
H	2.960092	0.079884	-0.977053
H	0.935187	-1.821442	-1.682518
H	0.712873	-1.144107	2.282681
H	-2.953176	-0.992008	0.344457
H	0.415767	1.195538	-2.336786
H	-0.712873	1.144107	2.282681

Transition state

Cu	-0.000000	0.000000	0.013390
N	1.590127	0.026852	-1.668416
N	1.674842	-0.020915	1.587549
N	-1.674842	0.020915	1.587549
N	-1.590127	-0.026852	-1.668416
N	0.000000	2.032650	0.064335
N	-0.000000	-2.032650	0.064335
H	2.547512	0.168791	-1.342832
H	1.360830	-0.192218	2.543886
H	-2.378844	0.740364	1.416870
H	-1.438268	-0.766439	-2.355660
H	1.635577	-0.824046	-2.230847
H	2.211292	0.845785	1.647632
H	-2.211292	-0.845785	1.647632
H	-2.547512	-0.168791	-1.342832
H	-0.001223	2.396701	1.018096
H	-0.813470	-2.452317	-0.387703
H	0.812243	-2.454208	-0.387876
H	-0.812243	2.454208	-0.387876
H	2.378844	-0.740364	1.416870
H	-1.360830	0.192218	2.543886
H	0.813470	2.452317	-0.387703
H	-1.635577	0.824046	-2.230847
H	0.001223	-2.396701	1.018096
H	1.438268	0.766439	-2.355660

$Cu(biea)_2$

Reactant/Product

Cu	0.068811	0.000001	-0.000000
N	-0.498153	-2.308588	0.000009
C	1.693566	-0.000009	-2.272337
N	0.423298	0.000009	1.997071
N	2.100920	0.000001	0.000000
N	-1.993181	-0.000002	-0.000000
N	-0.498159	2.308587	-0.000009
N	0.423298	-0.000007	-1.997071
C	1.693566	0.000011	2.272337
C	2.639988	0.000006	1.205960
C	2.639989	-0.000005	-1.205960
C	-2.594555	-1.180116	0.000004
H	-3.683710	-1.249731	0.000005
H	-3.683714	1.249721	-0.000004
C	-2.594559	1.180110	-0.000004
C	-1.793511	2.361574	-0.000009
C	-1.793504	-2.361578	0.000009
H	3.716346	-0.000006	-1.374380
H	3.716346	0.000007	1.374381
H	-0.205344	0.000015	2.792393
H	2.056416	0.000017	3.303966
H	2.056417	-0.000014	-3.303965
H	-0.205343	-0.000011	-2.792393
H	-0.068598	3.230548	-0.000013
H	-2.338900	3.313979	-0.000013
H	-2.338891	-3.313984	0.000013
H	-0.068589	-3.230548	0.000012

Transition state

Cu	0.000000	0.000000	0.000000
N	-0.000000	2.164043	0.438084
C	2.324560	-0.000000	-1.725248
N	-2.164043	-0.000000	-0.438084
N	0.000000	0.000000	-2.009560
N	0.000000	0.000000	2.009560
N	0.000000	-2.164043	0.438084
N	2.164043	0.000000	-0.438084
C	-2.324560	0.000000	-1.725248
C	-1.190659	-0.000000	-2.589474
C	1.190659	0.000000	-2.589474
C	-0.000000	1.190659	2.589474
H	0.000000	1.294257	3.674381
H	-0.000000	-1.294257	3.674381
C	0.000000	-1.190659	2.589474
C	-0.000000	-2.324560	1.725248
C	0.000000	2.324560	1.725248
H	1.294257	-0.000000	-3.674381
H	-1.294257	0.000000	-3.674381
H	-3.031832	0.000000	0.089763
H	-3.316492	0.000002	-2.190887
H	3.316492	-0.000002	-2.190887
H	3.031832	-0.000000	0.089763
H	-0.000000	-3.031832	-0.089763
H	-0.000002	-3.316492	2.190887
H	0.000002	3.316492	2.190887
H	0.000000	3.031832	-0.089763

Cu(timm)$_2^{2+}$

Reactant/Product

Cu	0.000000	0.000000	0.000000
H	−3.696636	−2.139409	0.000000
N	0.000000	−1.481347	1.379431
N	0.000000	1.481347	1.379431
N	0.000000	−1.481347	−1.379431
N	−2.407798	−0.492604	0.000000
H	0.535813	−1.434741	−2.246616
N	0.000000	1.481347	−1.379431
C	−0.689762	−2.540610	1.240661
H	0.706428	3.325230	−2.000941
H	−0.535813	1.434741	−2.246616
H	0.706428	3.325230	2.000941
C	−0.689762	−2.540610	−1.240661
H	0.535813	−1.434741	2.246616
H	−3.239291	0.101496	0.000000
C	0.689762	2.540610	−1.240661
H	−0.706428	−3.325230	2.000941
C	0.689762	2.540610	1.240661
H	−0.706428	−3.325230	−2.000941
N	2.407798	0.492604	0.000000
H	−0.535813	1.434741	2.246616
H	3.239291	−0.101496	0.000000
C	−2.683888	−1.726169	0.000000
H	3.696636	2.139409	0.000000
C	2.683888	1.726169	0.000000
C	−1.530514	−2.746453	0.000000
H	−1.941944	−3.757800	0.000000
C	1.530514	2.746453	0.000000
H	1.941944	3.757800	0.000000

Transition state

Cu	0.000000	0.000000	0.000000
H	0.191014	3.637880	1.884822
N	−1.820247	0.792983	−0.000000
N	−0.454898	−1.614046	1.466180
N	0.454898	1.614046	−1.466180
N	0.454898	1.614046	1.466180
H	1.020733	1.552543	−2.314130
N	1.820247	−0.792983	−0.000000
C	−2.024309	2.048830	−0.000000
H	3.033502	−2.466994	0.000000
H	2.664694	−0.220529	−0.000000
H	−0.191014	−3.637880	1.884822
C	0.000000	2.775312	−1.240615
H	−2.664694	0.220529	−0.000000
H	1.020733	1.552543	2.314130
C	2.024309	−2.048830	−0.000000
H	−3.033502	2.466994	0.000000
C	−0.000000	−2.775312	1.240615
H	0.191014	3.637880	−1.884822
N	−0.454898	−1.614046	−1.466180
H	−1.020733	−1.552543	2.314130
H	−1.020733	−1.552543	−2.314130
C	0.000000	2.775312	1.240615
H	−0.191014	−3.637880	−1.884822
C	−0.000000	−2.775312	−1.240615
C	−0.859400	3.011552	−0.000000
H	−1.230979	4.038006	−0.000000
C	0.859400	−3.011552	−0.000000
H	1.230979	−4.038006	−0.000000

Cu(ein)$_3^{2+}$

Reactant/Product

Cu	0.000000	−0.000000	0.076293
N	−0.797312	1.014358	1.661075
N	1.773013	1.600606	−0.337673
N	0.707536	−1.294483	−1.322030
N	−1.773013	−1.600606	−0.337673
N	0.797312	−1.014358	1.661075
N	−0.707536	1.294483	−1.322030
C	−0.000000	2.266325	−1.749014
C	1.367335	2.456774	−1.186804
C	−0.461780	0.583241	2.812702
C	0.461780	−0.583241	2.812702
C	−1.367335	−2.456774	−1.186804
C	−0.000000	−2.266325	−1.749014
H	−0.361465	2.961096	−2.509951
H	1.948477	3.315739	−1.531028
H	−0.810312	1.017427	3.751018
H	0.810312	−1.017427	3.751018
H	−1.948477	−3.315739	−1.531028
H	0.361465	−2.961096	−2.509951
H	1.629609	−1.215355	−1.752081
H	1.432414	−1.813302	1.659434
H	2.715305	1.799551	0.005449
H	−1.629609	1.215355	−1.752081
H	−1.432414	1.813302	1.659434
H	−2.715305	−1.799551	0.005449

Transition state

Cu	0.000000	−0.000000	0.058243
N	1.606778	0.405436	1.540660
N	−1.606778	−0.405436	1.540660
N	−1.338468	0.017160	−1.737218
N	1.338468	−0.017160	−1.737218
N	−0.000000	1.990105	0.204996
N	0.000000	−1.990105	0.204996
C	−0.827676	−2.549236	0.998702
C	−1.739248	−1.652631	1.760578
C	1.739248	1.652631	1.760578
C	0.827676	2.549236	0.998702
C	0.746666	0.008625	−2.863821
C	−0.746666	−0.008625	−2.863821
H	−0.884568	−3.631051	1.130384
H	−2.461382	−2.086604	2.455165
H	2.461382	2.086604	2.455165
H	0.884568	3.631051	1.130384
H	1.267527	0.041223	−3.823500
H	−1.267527	−0.041223	−3.823500
H	−2.357008	−0.004454	−1.809994
H	−0.613725	2.623223	−0.308292
H	−2.245298	0.182201	2.079900
H	0.613725	−2.623223	−0.308292
H	2.245298	−0.182201	2.079900
H	2.357008	0.004454	−1.809994

$Cu(en)_3^{2+}$

Reactant/Product				Transition state			
Cu	-0.000000	-0.000000	0.059806	Cu	-0.000000	-0.000000	0.055138
N	-0.000000	2.467823	-0.286936	N	-1.062646	1.289066	1.602798
N	-1.365484	-0.008846	1.626848	N	1.062646	-1.289066	1.602798
N	0.000000	-2.467823	-0.286936	N	0.672462	-1.265979	-1.742435
N	1.508204	-0.249787	-1.355093	N	-0.672462	1.265979	-1.742435
N	-1.508204	0.249787	-1.355093	N	-1.764841	-1.005861	0.169623
N	1.365484	0.008846	1.626848	N	1.764841	1.005861	0.169623
C	0.675129	-0.342028	2.890640	C	2.825874	0.193396	0.816479
C	-0.675129	0.342028	2.890640	C	2.243505	-0.508314	2.025987
C	-0.824416	2.614944	-1.499555	C	-2.243505	0.508314	2.025987
C	-1.993951	1.649893	-1.451935	C	-2.825874	-0.193396	0.816479
C	1.993951	-1.649893	-1.451935	C	0.000000	0.759073	-2.956372
C	0.824416	-2.614944	-1.499555	C	-0.000000	-0.759073	-2.956372
H	1.737088	0.957187	1.700965	H	1.617067	1.853957	0.718655
H	1.261356	-0.054529	3.769946	H	3.683469	0.813485	1.098981
H	0.553156	-1.430286	2.915934	H	3.180591	-0.540460	0.084459
H	-0.553156	1.430286	2.915934	H	1.916492	0.228209	2.768870
H	-1.261356	0.054529	3.769946	H	3.009452	-1.128690	2.505483
H	-1.737088	-0.957187	1.700965	H	1.378634	-2.160248	1.175543
H	0.881065	2.961393	-0.425198	H	-1.378634	2.160248	1.175543
H	-1.205782	3.634415	-1.636071	H	-3.009452	1.128690	2.505483
H	-2.614917	1.853669	-0.572057	H	-1.916492	-0.228209	2.768870
H	-0.195965	2.398328	-2.372313	H	-3.180591	0.540460	0.084459
H	-2.633733	1.781506	-2.331637	H	-3.683469	-0.813485	1.098981
H	-2.295805	-0.364673	-1.147815	H	-1.617067	-1.853957	0.718655
H	2.295805	0.364673	-1.147815	H	-0.486826	2.264340	-1.650686
H	2.614917	-1.853669	-0.572057	H	1.031354	1.132665	-2.959537
H	2.633733	-1.781506	-2.331637	H	-0.469681	1.129694	-3.874928
H	1.205782	-3.634415	-1.636071	H	0.469681	-1.129694	-3.874928
H	0.195965	-2.398328	-2.372313	H	-1.031354	-1.132665	-2.959537
H	-0.881065	-2.961393	-0.425198	H	0.486826	-2.264340	-1.650686
H	-1.197297	-0.049236	-2.279946	H	-2.094522	-1.335581	-0.737447
H	-2.180535	0.591601	1.505978	H	0.542563	-1.579621	2.430057
H	0.451259	-2.972390	0.476400	H	1.681276	-1.211667	-1.885413
H	2.180535	-0.591601	1.505978	H	2.094522	1.335581	-0.737447
H	-0.451259	2.972390	0.476400	H	-0.542563	1.579621	2.430057
H	1.197297	0.049236	-2.279946	H	-1.681276	1.211667	-1.885413

$Cu(tach)_2^{2+}$

Reactant/Product

Cu	0.000000	-0.000000	-0.000000
H	-3.644730	2.283825	0.000001
N	-1.236283	-0.821858	1.464357
N	1.236282	0.821853	1.464359
N	-1.236282	-0.821855	-1.464359
N	-1.562971	1.884610	0.000003
H	-1.050611	-0.369756	-2.360472
N	1.236282	0.821860	-1.464356
C	-2.717047	-0.817598	1.272914
H	3.162824	1.347183	-2.124857
H	0.941669	1.790746	-1.599508
H	3.162825	1.347177	2.124861
C	-2.717045	-0.817595	-1.272916
H	-0.941670	-1.790743	1.599513
H	-1.448274	2.505429	-0.802709
C	2.717046	0.817599	-1.272914
H	-3.162825	-1.347182	2.124857
C	2.717046	0.817594	1.272916
H	-3.162823	-1.347178	-2.124861
N	1.562971	-1.884609	-0.000002
H	0.941668	1.790739	1.599515
H	1.448274	-2.505430	-0.802712
C	-2.971971	1.415341	0.000001
H	3.644730	-2.283825	-0.000005
C	2.971971	-1.415341	-0.000002
C	-3.270543	0.606145	-1.262910
H	-4.359833	0.528863	-1.364576
C	3.083368	1.570574	0.000002
H	4.167594	1.732366	0.000002
C	-3.270545	0.606143	1.262910
C	-3.083368	-1.570574	-0.000002
H	-2.937034	1.154590	2.155500
H	-4.359835	0.528860	1.364574
H	-2.637213	-2.575407	-0.000003
H	-4.167594	-1.732366	-0.000003
H	-2.937030	1.154594	-2.155499
H	-0.941668	-1.790742	-1.599512
H	-1.448275	2.505428	0.802716
H	-1.050612	-0.369755	2.360469
C	3.270543	-0.606142	-1.262912
C	3.270545	-0.606146	1.262909
H	2.937034	-1.154595	2.155498
H	4.359833	-0.528860	-1.364577
H	4.359835	-0.528863	1.364573
H	2.937030	-1.154588	-2.155501
H	2.637213	2.575407	0.000004
H	1.050610	0.369761	-2.360470
H	1.050612	0.369752	2.360472
H	1.448274	-2.505425	0.802712

Transition state

Cu	0.000000	0.000000	0.000000
H	1.050883	3.452803	2.118668
N	-1.682649	1.152455	0.000000
N	-0.879877	-1.426424	1.538231
N	0.879877	1.426424	-1.538231
N	0.879877	1.426424	1.538231
H	1.890694	1.310016	-1.623944
N	1.682649	-1.152455	0.000000
C	-1.579326	2.643472	0.000000
H	2.603084	-3.039387	-0.000000
H	2.246728	-0.884950	0.807976
H	-1.050883	-3.452803	2.118668
C	0.622520	2.871122	-1.291518
H	-2.246728	0.884950	-0.807976
H	1.890694	1.310016	1.623944
C	1.579326	-2.643472	0.000000
H	-2.603084	3.039387	-0.000000
C	-0.622520	-2.871122	1.291518
H	1.050883	3.452803	-2.118668
N	-0.879877	-1.426424	-1.538231
H	-0.525029	-1.204743	2.469875
H	-0.525029	-1.204743	-2.469875
C	0.622520	2.871122	1.291518
H	-1.050883	-3.452803	-2.118668
C	-0.622520	-2.871122	-1.291518
C	1.297474	3.333012	0.000000
H	1.295771	4.429779	0.000000
C	0.879877	-3.138913	1.261792
H	1.034588	-4.222779	1.321435
C	-0.879877	3.138913	1.261792
C	-0.879877	3.138913	-1.261792
H	-1.362736	2.726874	2.159505
H	-1.034588	4.222779	1.321435
H	-1.362736	2.726874	-2.159505
H	-1.034588	4.222779	-1.321435
H	2.359319	3.047847	0.000000
H	0.525029	1.204743	-2.469875
H	0.525029	1.204743	2.469875
H	-2.246728	0.884950	0.807976
C	0.879877	-3.138913	-1.261792
C	-1.297474	-3.333012	0.000000
H	-2.359319	-3.047847	0.000000
H	1.034588	-4.222779	-1.321435
H	-1.295771	-4.429779	0.000000
H	1.362736	-2.726874	-2.159505
H	1.362736	-2.726874	2.159505
H	2.246728	-0.884950	-0.807976
H	-1.890694	-1.310016	1.623944
H	-1.890694	-1.310016	-1.623944

References

1 W. Jiang, N. J. DeYonker, J. J. Determan and A. K. Wilson, *J Phys Chem A*, 2012, **116**, 870–885.
2 A. Karton, E. Rabinovich, J. M. L. Martin and B. Ruscic, *J. Chem. Phys.*, 2006, **125**, 144108.
3 I. Bertini, D. Gatteschi and A. Scozzafava, *Inorg. Chem.*, 1977, **16**, 1973–1976.
